# Implications of Physical Activity in Reducing Alcohol Use for Women Veterans: A Narrative Review

**DOI:** 10.1089/whr.2023.0188

**Published:** 2024-07-08

**Authors:** Alfredo Gil, Donna Schuman, Keng-Yu Chang, Zhaoli Liu, Chueh-Lung Hwang

**Affiliations:** ^1^ Department of Kinesiology, The University of Texas at Arlington, Arlington, Texas, USA.; ^2^ School of Social Work, The University of Texas at Arlington, Arlington, Texas, USA.; ^3^ College of Nursing and Health Innovation, The University of Texas at Arlington, Arlington, Texas, USA.

**Keywords:** binge drinking, exercise, minority health, health disparity, women veterans

## Abstract

Women veterans (WV) are a fast-growing population in the United States with concerning health disparities. Reports of increased risks of cardiovascular disease (CVD) and poorer health are evident in WV compared with their civilian counterparts. The transition from active duty to veteran poses additional life stressors, causing changes in health behaviors such as unhealthy alcohol consumption and decreased physical activity, which may explain health disparities in WV. The changes in these two health risk behaviors may be influenced by each other, and emerging evidence suggests that physical activity aids in managing alcohol consumption during alcohol use treatment. In this general narrative review, we summarized findings from studies involving WV on (1) the associations between alcohol consumption and physical activity and (2) the effect of physical activity on reducing alcohol use. We also discussed the clinical consideration of adding physical activity to alcohol use interventions for WV. Most of the literature included in this review has been based on predominantly veteran men populations. This knowledge gap highlights the importance of continued efforts and research studies targeting WV to eliminate health disparities among them.

## Introduction

With 17.3% of the U.S. military community being women, Women veterans (WV) are expected to number over 2 million in 2025.^1^ This fast-growing population is experiencing several multifaceted and interconnected health problems, predisposing WV to cardiovascular disease (CVD).^2–4^ In U.S. veterans, CVD remains one of the leading causes of death.^5^ Indeed, veteran status itself is reported as a risk factor of CVD.^2^ Compared with male veterans, WV may have an increased risk for CVD because of their unique military service experience, sociodemographic, and/or changes in health behaviors after separating from the military.^3^ The transition from active duty to veteran status poses many lifestyle changes which may cause emotional distress and thereby the development of negative health behaviors, such as unhealthy alcohol consumption and decreased physical activity.^4,6^ Emerging evidence suggests that these two health risk behaviors may be related to each other. However, while increasing physical activity is known to provide several health benefits and is recommended to reduce risks for CVD,^3,7^ alcohol use has received minimal attention in CVD care for WV.

### Alcohol use in women veterans

Alcohol is the most common form of substance use in veterans.^8,9^ Compared with civilian women, WV are more likely to consume alcohol in their lifetime.^10,11^ A recent study using data from the 2002–2019 National Study on Drug Use and Health found that veteran status in women was associated with more frequent and heavier drinking.^12^ In veterans, unhealthy alcohol use is associated with an increased risk of all-cause mortality and CVD^10,13,14^ and poor health-related quality of life.^15^

The National Institute on Alcohol Abuse and Alcoholism recommends that for health considerations, alcohol consumption for women should be no more than one drink per day, such as a 12 fl oz of regular beer (5% alc/vol) or 5 oz of wine (12% alc/vol). Binge drinking, the most common pattern of excessive alcohol use, is defined as alcohol consumption of 4 drinks or more on any occasion for women. A systematic review shows that binge drinking rates in WV ranged from 7% to 37%,^16^ while data from the 2021 National Survey of Drug Use and Health show that in general population, 20.9% of women aged 18 and older reported binge drinking in the past 30 days.^17^ These binge drinking rates are potentially underestimated as WV who are at risk of unhealthy alcohol use usually do not utilize the Veterans Health Administration and are less likely to be included in research studies.^18^ Although trends in WV may be inadequately represented in studies, data from the Department of Defense Health Related Behaviors Survey (HRBS) show that binge drinking rates increased in active-duty service women by 18% from 2015 to 28.2% in 2018.^19^ With the increasing rates of binge drinking in active-duty service women,^19^ it is possible that the prevalence of binge drinking would be expected to also increase as WV transition into civilian life.

### Contributing factors of alcohol use in WV

Emotional distress during the transition from active duty to civilian life may cause unhealthy drinking behaviors in WV.^4^ Schreiner et al. found that veterans (14% WV) continued to consume alcoholic beverages for facilitating social interactions, improving sleep, and relieving tension.^20^ Creech and Borsari found that WV who used alcohol drinking as a coping strategy to avoid emotional distress were at greater risk of binge and heavy drinking.^21^ These unhealthy drinking behaviors in WV were also associated with a higher degree of individual positive outcome expectancies from drinking—the belief that alcohol would bring to them positive effects on a variety of domains, such as sociability and tension reduction.^21^ In addition, unhealthy drinking behaviors in WV are attributable to poor mental health, including hostility,^22^ depression, posttraumatic stress disorder (PTSD),^23^ experience of intimate partner violence,^24^ military sexual trauma, and combat exposure.^25^ Compared with veteran men, WV have a higher rate of military-related sexual trauma, which increases the risks of PTSD, depression, anxiety, and alcohol and other substance use disorders.^26^

### Physical activity in substance use treatment

The physical activity guidelines for Americans recommends that adults should perform 150 minutes to 300 minutes (or 75 minutes to 150 minutes) of moderate-intensity (or vigorous-intensity) aerobic physical activity, plus at least 2 days of muscle-strengthening activity a week to maintain health.^27^ While physical activity is known to moderate CVD risk and occurrence,^27^ emerging evidence supports the role of physical activity in substance use treatment. Findings from a meta-analysis of 22 randomized controlled studies showed that exercise, a form of physical activity, increased alcohol abstinence and eased withdrawal symptoms in adults.^28^ In another randomized controlled study, Brown et al. found that aerobic exercise, as an adjunct to behavioral treatment for alcohol use, reduced drinking days and heavy drinking days in adults with alcohol use disorder.^29^

Increasing physical activity in veterans has been shown to improve physical and cognitive function,^30^ promote weight loss,^31^ reduce pain, and improve sleep,^32^ all of which are associated with unhealthy drinking behaviors in veterans. Therefore, physical activity seems to be a promising strategy to help reduce alcohol use in WV. Although WV have been reported to have a higher level of physical activity, there were no differences in the percentage of meeting guidelines for aerobic physical activity between WV (51.9%) and civilian women (49.5%).^33^ In addition, physical activity in WV declines after military service,^4,6^ and WV may have a greater decrease in physical activity every year than civilian women.^33^ These further highlight the need for physical activity involvement in WV. This narrative literature review will provide a summary of the current evidence on 1) the association between physical activity and alcohol use; and 2) the effect of physical activity on reducing alcohol use in WV.

## Methods

To conduct this review, we used the 6-step approach from Machi and McEvoy^34^ that includes the following steps: Select a Topic, Develop Tools of Argumentation, Search the Literature, Survey the Literature, Critique the Literature, and Write the review. Some of these steps are described below.

### Search and survey the literature

A literature search was conducted by A.G. in consultation with D.S. and C.L.H. using the two electronic databases: PubMed and PsycINFO (March 5, 2024) and the following search terms and strategies: (physical activity or exercise), (alcohol use, alcohol drinking, drinking, or substance use), and (veterans, WV, or military). Studies were included in this review if the following inclusion criteria were met: (1) studies included WV; (2) any original studies investigated the correlation between alcohol use and physical activity or studies investigated the effect of physical activity intervention on alcohol use; and 3) studies were published in 2000 or after. Studies were excluded from this review if the studies did not meet all these inclusion criteria. After the initial review of titles and abstracts, the full‐text review was performed independently by the authors, A.G. and C.L.H. Any discrepancy in inclusion decision was discussed with the authors, D.S. and Z.L.L., for the final inclusion decisions.

### Data extraction

Data were extracted by the authors A.G. and K.Y.C. for each included study and confirmed by the author C.L.H. Primary outcomes for this review were: (1) alcohol consumption including the assessment tools and definition of drinking categories (*e.g.,* how many drinks per day, how many drinking days per week, and/or length of drinking history); and (2) physical activity including the assessment tools and type (*e.g.,* walking exercise or yoga), intensity (*e.g.,* moderate intensity), duration (*e.g.,* 30 min each time), frequency (*e.g.,* three times per week) and/or length (*i.e.,* past year history or 8-week intervention) of physical activity. Subject number, characteristics, such as age and clinical characteristics, were also extracted from the reviewed studies.

### Critique the literature

The National Heart, Lung, and Blood Institute Study Quality Assessment Tools were used to evaluate the quality of the included studies and identify potential publication bias. This tool consists of different items/questions specific to the study design of the reviewed study regarding hypothesis/objectives, inclusion/exclusion criteria of study participants, outcome measures, sample size, and statistical analysis *etc.* These items/questions are listed as the footnotes in Table 3 and 4 specific to the study design of the reviewed study.

## Results

### Study selection

We identified 151 articles from PubMed and 113 articles from PsycINFO. After cross-checking the repeated records, a total of 240 articles were included for title and abstract review and out of these articles, 46 articles were selected for full-text review to confirm inclusion eligibility. Seven studies were included in this review after excluding articles that were (1) irrelevant to our topic of interest (*n* = 31; *i.e.,* no report of association between alcohol use and physical activity, no report of alcohol use assessment, no description of physical activity intervention *etc.*); (2) did not include WV (*n* = 3); and (3) were either book chapters, dissertation, or formative research (*n* = 5).^30,35–40^ One article^41^ was identified from the list of references in one published review.^42^ Therefore, a total of eight studies were included in this narrative review. Five studies reported data on the association between alcohol consumption and physical activity behaviors,^35–37,39,40^ and three studies investigated the effect of physical activity on reducing alcohol use in WV.^30,38,41^

### Synthesis of study findings

#### Association between alcohol consumption and physical activity behaviors (n = 5)

A total of five studies were included in this review to investigate the association between alcohol consumption and physical activity behaviors in WV (Table 1). Except for one study with a sample size of 200,^36^ other studies included participants ranging from 1,140 to 185,323.^35,37,39,40^ These studies were predominately in veteran men (2–11% WV). One study included veterans with PTSD, 51% of which were WV.^39^ Two studies included participants from the Million Veteran Program and categorized alcohol use and physical activity using the program-specific self-reported questionnaire.^35,37^ Among the remaining three studies, one study used a single multiple choice questionnaire to categorize alcohol use,^36^ while the other two used the Alcohol Use Disorder Identification Test (AUDIT).^39,40^ Two studies used a single-item question to categorize/define physical activity,^36,39^ while one study conducted latent class analysis to identify exercise class from the related non-pharmacological health practice.^40^

**Table 1.** Summary of the Included Studies Including Women Veterans on the Associations between Alcohol Consumption and Physical Activity Behaviors (*n* = 5)

Authors; study design to the outcomes of interests	Participant characteristics	Assessment		
		Alcohol use	PA	Findings
Donaldson et al., 2018; Population-based cross-sectional analysis of cohort-study data^40^	U.S. veterans with combat deployments from the Readiness and Resilience in National Guard Soldiers (RINGS) longitudinal cohort study *n* = 1,850 (10% WV) Age 39 ± 9 yr	AUDIT Problem drinking: AUDIT score ≥8	Self-reported past-year use of Health Practices Inventory survey of 19 health-related practice PA category identified by latent class analysis: exercise class versus low use of health practice class; exercise class had a high rate of strength/stretch exercise (86%) and aerobic exercise (86%)	Compared with low use class, exercise class had a similar prevalence of problem drinking.
Jazieh et al., 2006; Sample-based cross-sectional study^36^	U.S. veterans diagnosed with m alignancy *n* = 200 (2% WV) Age NR; 60.5% >65 yr	Single self-reported multiple-choice question Drinking pattern after cancer diagnosis: increased, decreased, or remained the same	Single self-reported multiple-choice question PA pattern after cancer diagnosis: increased, decreased, or remained the same amount of exercise	No significant correlations between changes in exercise and changes in alcohol drinking after cancer diagnosis
Song et al., 2018; Population-based cross-sectional analysis of cohort-study data^35^	U.S. veterans free of prevalent CAD from the Million Veteran Program *n* = 156,728 (9%WV) Age 65 ± 12 yr	Self-reported food frequency questionnaire over the past year from the Lifestyle Survey Drinking categories: never, former, ≤6 g/day, >6–12 g/day, >12–24 g/day, >24–36 g/day, >36–48 g/day, and >48 g/day or AUD	Self-reported, from the Lifestyle survey PA category based on exercise frequency: ≤1 day/week, 2–4 days/week, and ≥5 days/week	Percentage data of exercise frequency were presented. See the Result section for details.
			One standard drink: 12 g of ethanol		
Song et al., 2023; Population-based cross-sectional analysis of cohort-study data^37^	U.S. veterans free of CVD or AUD from the Million Veteran Program *n* = 185,323 (11% WV) Age 64 ± 13 yr	Self-reported food frequency questionnaire over the past year from the Lifestyle Survey Drinking Category: never, former, <1 drink/day, 1–2 drinks/day, >2–3 drinks/day, and ≥3 drinks/day.	Self-reported, from the Lifestyle survey PA category based on exercise frequency: ≤1 day/week, 2–4 days/week, and ≥5 days/week	Compared with never drinkers, veterans who had 1–2 drinks/day had higher exercise frequency	
		No definition of one standard drink			
Whitworth et al., 2022; Population-based cross-sectional analysis of cohort-study data^39^	U.S. veterans with PTSD from Project VALOR (Veterans’ After discharge Longitudinal Registry) *n* = 1,140 (51% WV) Age 43 ± 10 yr	AUDIT Possible AUD: AUDIT score ≥8	Self-reported Rapid Physical Activity Questionnaire Physical inactivity determined by responding to a single item from “Yes” to “I rarely or never do any physical activities”	Physical inactivity was not significantly associated with a diagnosis of PTSD plus AUD	

Data are mean ± SD.

AUD, alcohol use disorder; AUDIT, Alcohol Use Disorders Identification test; CAD, coronary artery disease; CVD, cardiovascular disease; *N*, number of studies; *n,* number of study participants; PA, physical activity; PTSD, posttraumatic stress disorder; WV, women veterans.

Aside from the two studies by the Million Veteran Program,^35,37^ findings from the other studies did not suggest an association between alcohol consumption and physical activity.^36,39,40^ Using data from the Million Veteran Program, Song et al. reported that 35%−40% of current drinkers (with alcohol consumption ranging from ≤6 g/day to >36–48 g/day of ethanol; ∼9% WV) exercised 2–4 times per week, whereas the rates were 28% in lifetime alcohol abstainers (14% WV), 27% in former drinkers (9% WV), and 26% in those with a diagnosis of alcohol use disorder or alcohol consumption of >48 g per day (7% WV).^35^ The same author group further found that veterans consuming 1–2 drinks/day (6% WV)^35^ had higher exercise frequency than lifetime abstainers (14.7% WV).^37^ These findings suggest that veterans who are moderate drinkers engage in exercise more than veterans who do not drink.

#### Effect of physical activity interventions on reducing alcohol use (n = 3)

A total of three studies were included in this review to examine the effects of physical activity interventions on reducing alcohol use in WV (Table 2). The sample size in these studies ranged from 15 to 45 participants with varying populations of veterans and civilians. In two studies that only included veterans, the participants were undergoing treatment for alcohol/substance use disorder and predominately men (13%−28% WV). Another study of women with PTSD included both civilian women and WV (24%). Physical activity interventions used in these studies were all under supervision with a retention rate of at least 70%. These interventions included a variety of frequency, duration, type, and length and none of the studies reported intensity. To track the change in alcohol use after physical activity intervention, two studies used the timeline follow-back calendar and another study used AUDIT. Two out of the three studies found that physical activity interventions reduced alcohol use or risk of alcohol use, measured by drinking amount,^30,38^ drinking days,^38^ and alcohol craving.^30^ However, adding virtual reality into physical activity intervention did not result in further reduction in alcohol use and alcohol craving.^30^ On the other hand, Reddy et al., found no difference in the change in AUDIT score over time between yoga intervention and waitlist control period suggesting no change in alcohol use.^41^

**Table 2.** Summary of the Studies Including Women Veterans on the Effect of Physical Activity on Reducing Alcohol Use (*n* = 3)

Authors; study design	Participant characteristics	Alcohol use assessment	PA intervention	Alcohol use after intervention versus baseline
Linke et al., 2019; Single-arm pilot study^38^	US Veterans in the outpatient Alcohol & Drug Treatment Program EX: *n* = 15, 13% WV, age 45 ± 10 yr; 60% reported alcohol use (5 ± 5 drinks/day, 18 ± 9 drinking days/month)	TLFB	12-week the Go-VAR! (Veterans Active Recovery) program: 1. Weekly 90-min psychoeducation group sessions 2. YMCA gym membership and weekly group exercise 3. Fitbit Charge HR to motive daily exercise Retention rate: 73%	Drinking Days per month ↓ Drinks per day ↓
Pennington et al., 2022; Randomized adaptive^30^	US Veterans with traumatic brain injury who sought for AUD treatment and were identified as heavy drinkers EX: *n* = 15, 13% WV, age 49 ± 11 yr, alcohol use 28 ± 27 drinks/week, ADUIT score: 16.4 ± 7.8 CON: *n* = 15, 20% WV, age 53 ± 8 yr, alcohol use 20 ± 28 drinks/week, ADUIT score: 16.8 ± 8.2 EX+VR: *n* = 23 (from EX and CON), 22% WV, age 52 ± 10 yr, alcohol use 25 ± 26 drinks/week, ADUIT score: 16.1 ± 7.8	TLFB Obsessive Compulsive Drinking Scale	Phase 1: 3-week supervised intervention, 30 min/session, 3 times/week EX: stationary recumbent Bicycle, self-pace; retention rate: 73% CON: tablet-based gameplay only; retention rate: 80% Phase 2: One week after Phase 1, 3-week supervised “EX+VR path-based adventure game” intervention, 30 min/session, 3 times/week for those who completed the Phase 1; retention rate: 70%	Drinks per week and Obsessive Compulsive Drinking Scale ↓ in EX only
Reddy et al., 2014; Randomized controlled pilot^41^	U.S. women with PTSD EX: *n* = 20, 30% WV, age 46 ± 12 yr, ADUIT score 2.0 ± 2.3 CON: *n* = 18, 17% WV, age 43 ± 13 yr, ADUIT score 3.3 ± 4.6	AUDIT	EX: Kripalu-based yoga. 75 min/sessions, a total of 12 sessions either over 6 weeks or 12 weeks; retention rate: 70% CON: None; timeline matched with EX; retention rate: 61%	AUDIT↔

Data are mean ± SD.

AUD, alcohol use disorder; ADUIT, Alcohol Use Disorders Identification test; CON, control group; EX, experimental group; HR, heart rate; *N*, number of studies; *n*, number of study participants; PA, physical activity; PTSD, posttraumatic stress disorder; TLFB, timeline follow-back; VR, virtual reality; WV, women veterans; ↓, decreased; ↔, remained the same.

### Study quality

The five studies examining the associations between alcohol consumption and physical activity behaviors were either cross-sectional studies or cross-sectional analyses of cohort-study data (Table 1). In three of the five studies, the authors described the purposes/research questions related to the review question (Q1; Table 3).^36,39,40^ All studies clearly specified and defined the study population and participants were recruited (or included in the analysis) using the same inclusion and exclusion criteria (Q2 and Q4). Participation rates were overall low with one study reporting a response rate of 48.3% to study invitation for potential subjects^40^ while other studies experienced participation rate below 50% of eligible subjects.^35,37^ Two studies did not provide information on participation rates (Q3).^36,39^ Given the exploratory and cross-sectional nature of these studies, none of the authors provided sample size justification (Q5). However, most of the studies did include a large sample size.^35,37,39,40^ Alcohol use and physical activity assessments were only conducted at a single time point during the same timeframe (Q6, Q7, Q10, Q12, and Q13). Three studies categorized alcohol use and physical activity to different levels, while the other two studies used one cut-off to categorize alcohol use (Q8).^39,40^ All studies provided descriptions of alcohol use and physical activity assessments; however, data relied on the use of self-report only (Q9, Q11). Two studies controlled potential confounding variables to examine the association between alcohol consumption and physical activity behaviors (Q14).^39,40^

**Table 3.** Quality Assessment of Included Studies Including Women Veterans on the Associations between Alcohol Consumption and Physical Activity Behaviors Using the Modified Study Quality Assessment Tools from the National Heart, Lung, and Blood Institute for Observational Cohort and Cross-Sectional Studies (*n* = 5)

Authors	Q1^a^	Q2^b^	Q3^c^	Q4^d^	Q5^e^	Q6^f^	Q7^g^	Q8^h^	Q9^i^	Q10^j^	Q11^k^	Q12^l^	Q13^m^	Q14^n^
Donaldson et al., 2018^40^	Yes	Yes	No	Yes	No	No	No	No	Maybe	No	Maybe	No	NA	Yes
Jazieh et al., 2006^36^	Yes	Yes	NR	Yes	No	No	No	Yes	Maybe	No	Maybe	No	NA	No
Song et al., 2018^35^	No	No	No^51^	Yes	No	No	No	Yes	Maybe	No	Maybe	No	NA	No
Song et al., 2023^37^	No	No	No^51^	Yes	No	No	No	Yes	Maybe	No	Maybe	No	NA	No
Whitworth et al., 2022^39^	Yes	Yes	NR	Yes	No	No	No	No	Maybe	No	Maybe	No	NA	Yes

List of questions: ^a^Was the research question or objective in this article related to the review question? (modified from the original question) ^b^Was the study population clearly specified and defined? ^c^Was the participation rate of eligible persons at least 50%? ^d^Were all the subjects selected or recruited from the same or similar populations (including the same time period)? Were inclusion and exclusion criteria for being in the study prespecified and applied uniformly to all participants? ^e^Was a sample size justification, power description, or variance and effect estimates provided? ^f^For the analyses in this article, were the exposure(s) of interest measured prior to the outcome(s) being measured? ^g^Was the timeframe sufficient so that one could reasonably expect to see an association between exposure and outcome if it existed? ^h^For exposures that can vary in amount or level, did the study examine different levels of the exposure as related to the outcome (*e.g.,* categories of exposure, or exposure measured as continuous variable)? ^i^Were the exposure measures (alcohol use) clearly defined, valid, reliable, and implemented consistently across all study participants? ^j^Was the exposure(s) assessed more than once over time? ^k^Were the outcome measures (physical activity) clearly defined, valid, reliable, and implemented consistently across all study participants? ^l^Were the outcome assessors blinded to the exposure status of participants? ^m^Was loss to follow-up after baseline 20% or less? ^n^Were key potential confounding variables measured and adjusted statistically for their impact on the relationship between exposure(s) and outcome(s)?.

As for the three included studies investigating the effect of physical activity on reducing alcohol use, there was no control group in the study by Linke et al. and in phase 2 of the study by Pennington et al. (Table 1).^30,38^ These two studies (pre-post studies with no control group in Table 4) included clearly defined objectives, inclusion/exclusion criteria for participation, processes to determine eligibility, and a specific clinical population of interest (Q1, Q2, Q3, and Q4). Sample sizes were relatively small (Q5). The physical activity interventions conducted in these studies were described overall in detail but with no report of activity intensity (Q6). Alcohol use assessment was clearly defined but based solely on self-report questionnaires (Q7 and Q8). In these two studies, more than 20% of study participants did not complete the study, with one study excluding these subjects from the statistical analysis^38^ and one including them (Q9).^30^ Alcohol use was compared before and after the intervention using statistical analysis with a report of mean, *SD*, and *p* values (Q10 and Q11).

**Table 4.** Quality Assessment of Included Studies Including Women Veterans on the Effect of Physical Activity on Reducing Alcohol Use Using the Modified Study Quality Assessment Tools from the National Heart, Lung, and Blood Institute for before-after (Pre-Post) Studies with No Control Group (*n* = 2) and for Controlled Intervention Studies (*n* = 2)

	List of questions													
Authors	Q1^a^	Q2^b^	Q3^c^	Q4^d^	Q5^e^	Q6^f^	Q7^g^	Q8^h^	Q9^i^	Q10^j^	Q11^k^	Q12^l^		
Before-after (Pre-Post) Studies with No Control Group														
Linke et al., 2019^38^	Yes	Yes	Maybe	Yes	No	No	Maybe	No	No	Yes	No	NA	—	—
Pennington et al., 2022 (Phase 2)^30^	Yes	Yes	Maybe	Yes	No	No	Maybe	No	No	Yes	Yes	NA	—	—

	**Q1^1^**	**Q2^2^**	**Q3^3^**	**Q4^4^**	**Q5^5^**	**Q6^6^**	**Q7^7^**	**Q8^8^**	**Q9^9^**	**Q10^10^**	**Q11^11^**	**Q12^12^**	**Q13^13^**	**Q14^14^**
Controlled Intervention Studies														
Pennington et al., 2022 (Phase 1)^30^	Yes	Yes	CD	No	No	Yes	No	Yes	Yes	CD	Yes	No	No	Yes
Reddy et al., 2014^41^	Yes	Yes		CD	No	Yes	No	Yes	NR	CD	No	No	Yes	Yes

List of questions for before-after (pre-post) studies with no control group: ^a^Was the study question or objective clearly stated? ^b^Were eligibility/selection criteria for the study population prespecified and clearly described? ^c^Were the participants in the study representative of those who would be eligible for the test/service/intervention in the general or clinical population of interest? ^d^Were all eligible participants that met the prespecified entry criteria enrolled? ^e^Was the sample size sufficiently large to provide confidence in the findings related to alcohol reduction? ^f^Was the test/service/intervention clearly described and delivered consistently across the study population? ^g^Were the outcome measures (alcohol use) prespecified, clearly defined, valid, reliable, and assessed consistently across all study participants? ^h^Were the people assessing the outcomes blinded to the participants' exposures/interventions? ^i^Was the loss to follow-up after baseline 20% or less? Were those lost to follow-up accounted for in the analysis? ^j^Did the statistical methods examine changes in outcome measures from before to after the intervention? Were statistical tests done that provided p values for the pre-to-post changes? ^k^Were outcome measures of interest taken multiple times before the intervention and multiple times after the intervention (*i.e.,* did they use an interrupted time-series design)? ^l^If the intervention was conducted at a group level (*e.g.,* a whole hospital, a community, *etc.*) did the statistical analysis take into account the use of individual-level data to determine effects at the group level?.

List of questions for controlled intervention studies: ^1^Was the study described as randomized, a randomized trial, a randomized clinical trial, or an RCT? ^2^Was the method of randomization adequate (*i.e.,* use of randomly generated assignment)? ^3^Was the treatment allocation concealed (so that assignments could not be predicted)? ^4^Were study participants and providers blinded to treatment group assignment? ^5^Were the people assessing the outcomes blinded to the participants' group assignments? ^6^Were the groups similar at baseline on important characteristics that could affect outcomes (*e.g.,* demographics, risk factors, co-morbid conditions)?^7^Was the overall drop-out rate from the study at endpoint 20% or lower of the number allocated to treatment? ^8^Was the differential drop-out rate (between treatment groups) at endpoint 15 percentage points or lower? ^9^Was there high adherence (>70%) to the intervention protocols for each treatment group? ^10^Were other interventions avoided or similar in the groups (*e.g.,* similar background treatments)? ^11^Were outcomes (alcohol use) assessed using valid and reliable measures, implemented consistently across all study participants? ^12^Did the authors report that the sample size was sufficiently large to be able to detect a difference in the main outcome between groups with at least 80% power? ^13^Were outcomes reported or subgroups analyzed prespecified (*i.e.,* identified before analyses were conducted)? ^14^Were all randomized participants analyzed in the group to which they were originally assigned, *i.e.,* did they use an intention-to-treat analysis?.

CD, cannot determine.

In another two studies with a control group (controlled Intervention Studies in Table 4),^30,41^ the authors used a randomized design with an adequate description of randomization (Table 1 and Q1 and Q2 in Table 4). However, no information was found in either of these studies to determine whether the allocation was concealed or not (Q3). The participants, intervention providers, and outcome assessors (self-report questionnaires) were all aware of the group allocation (Q4 and Q5). Within the same studies, participants in the intervention and control groups had similar baseline measures and dropout rates (Q6 and Q8) with a dropout rate over 20% for both interventions (Q7). Neither study reported a power calculation (Q12). The adherence rate to the intervention was higher than 70% in one study,^30^ but unreported in another (Q9).^41^ It is unclear whether participants in the studies received any other interventions which may influence alcohol use outside of the study (Q10). One study used AUDIT, which captures past-year alcohol use, and may not be reliable to track a change in alcohol use during the study (12 weeks; Q11).^41^ The primary outcome of one study was to determine feasibility and to collect preliminary data for hypothesis generation (Q13).^30^ Lastly, both studies conducted an intention-to-treat analysis with one using a linear mixed model (Q14).^41^

## Discussion

To our knowledge, this is the first narrative review on the association of physical activity to alcohol use and the effect of physical activity on reducing alcohol use in WV. Based on a limited number of included studies, the results of this review suggest that although the association between physical activity and alcohol use is equivocal, physical activity interventions may be beneficial in reducing alcohol use for WV.

### Association between alcohol consumption and physical activity behaviors

Our review results indicate that the association between alcohol consumption and physical activity in WV is equivocal. First, only a limited number of studies examined the association in veteran population with a poor to fair study quality and conflicting study findings. One weakness of these included studies is that alcohol consumption and physical activity were not the primary outcomes. In addition, alcohol and physical activity assessments/categories and population characteristics varied among studies. It also seemed that clinical characteristics, such as cancer^36^ and PTSD,^39^ may have masked the association between alcohol consumption and physical activity. Moreover, none of the studies measured “binge drinking” and all used simplified and self-reported physical activity measurements without considering different components of physical activity (*e.g.,* type, intensity, duration, and/or length). Lastly, there was a lack of studies targeting WV. Because of these limitations, we were unable to conclude the association between alcohol use and physical activity in the WV population.

Among the included studies, studies from the Million Veterans Program included the largest sample size (11% WV) and the findings suggest that alcohol consumption of 1–2 drinks/day is associated with a higher exercise frequency, or more physically active lifestyle.^37^ Similar associations have been previously reported in veteran men^43^ and in the general population.^44–46^ Rittmueller et al. collected mailed questionnaires from 11,927 veteran men with hypertension^43^ and found that compared to non-drinkers, veteran men with an AUDIT-C score of 4–5 were more likely to engage in regular exercise.^43^ The AUDIT-C score was calculated from the first three items from AUDIT and score of 4–5 is equivalent to the consumption up to 6 drinks per week and/or a history binge drinking less than monthly to monthly. Using data from the UK biobank of 371,463 adults aged 40–69 (54% women), Biddinger et al. found that compared to alcohol abstainers, current drinkers had more days per week of performing more than 10 minutes of moderate physical activity a day.^45^ Shuval et al. used data of 10,922 apparently healthy women drinkers from the Cooper Center Longitudinal Study. Their findings indicate that individuals with higher self-reported physical activity (equal to or higher than the activity guidelines) were more likely to consume alcohol at a moderate (>3–7 drinks per week) or heavy amount (>7 drinks per week) rather than a light amount (1–3 drinks per week), compared with those who did not meet activity guidelines.^46^ In addition, Smothers & Bertolucci, using data from the 1990 National Health Interview Survey,^44^ found that compared to lifetime alcohol abstainers, women drinkers were more likely to have a physically active lifestyle (defined as activities on at least 5 times per week and a total of at least 150 minutes per week). The association was more pronounced in moderate drinkers (4–7 drinks per week for women).^44^ Taken together, these study findings suggest that alcohol consumption and a physically active lifestyle may coexist in women, at least in the general population.^44–46^

### Effect of physical activity interventions on reducing alcohol use

Our review results suggest a potential role of physical activity in reducing alcohol use in WV who are heavy drinkers or with a diagnosis of alcohol use disorder. Linke et al. demonstrated that a multicomponent exercise-based intervention was feasible and effective for reducing alcohol use for veterans undergoing treatment for alcohol use disorders.^38^ Pennington et al. also reported a reduction in alcohol use following an exercise intervention in veterans with traumatic brain injury who sought treatment for alcohol use disorder and identified as heavy drinkers.^30^ In addition, along with a reduction in alcohol use, Pennington et al. reported improvements in physical activity (measured by daily steps) as well as physical and mental health.^38^ Kendzor et al. found that veteran men who were identified as heavy drinkers had a decline in physical activity following a 12-month alcohol reduction intervention.^47^ Although the authors reported that the decline in physical activity was not associated with the reduction in alcohol consumption, these findings suggest the importance of adding physical activity interventions into alcohol use programs.

Reddy et al. demonstrated no significant difference in the change in alcohol use, as measured by AUDIT, between a yoga intervention and a non-exercise control group among women civilians and WV with PTSD. Several factors may have contributed to insignificant findings. Primarily, most of the study participants were more likely to be alcohol abstainers or low-risk drinkers (mean AUDIT score 2.6 ± 3.6). Additionally, the lack of observed improvement in risks of alcohol use after yoga intervention may be because the intervention did not include alcohol abstinence/reduction. Lastly, AUDIT measures past-year alcohol use and may not be sensitive to detect changes in alcohol use over shorter intervals. Indeed, all the included studies tracked changes in alcohol use during physical activity intervention by using self-report questionnaires introducing recall bias. This is an issue given that study participants were aware of group allocation. Future studies can use biomarkers, such as dried blood spot phosphatidylethanol, to provide objective and reliable measures. Although none of these studies have sample sizes specifically powered to detect changes in alcohol use, they do provide the preliminary findings for sample size calculation. High-quality randomized controlled studies are warranted to determine the effect of physical activity interventions on reducing risks of alcohol use targeting WV.

### Clinical considerations and future directions

Veterans undergoing treatment for substance use disorder have been reported to be interested in participating in physical activities.^48^ Our review findings in veteran populations (primarily involving veteran men as participants) suggest that physical activity interventions can be a great addition to alcohol use treatment and facilitate the reduction in risks associated with alcohol use.^30,38^ Even if there is no interaction between physical activity and alcohol use, given that WV are at risk of CVD, there is still a need to develop interventions that include components to target alcohol use and physical activity respectively. Such integrated interventions aim to reduce negative behaviors (*i.e.,* alcohol consumption) and at the same time, promote favorable behaviors (*i.e.,* physical activity), thus maximizing health outcomes for WV.

There are several considerations for integrating physical activity into alcohol use interventions for WV. The transition from military to civilian life introduces significant environmental changes that play a key role in WV’s health. During active-duty service, women must maintain a standard level of physical performance to be physically prepared for deployment at any time. Once women service members are discharged or released, there are no standards to uphold and to motivate them to stay physically active or to avoid drinking excessively. These women also lose the immediate and free access to wellness programs they received on active duty. Therefore, it is important to initialize interventions early when women are still on active duty to facilitate their transition to veteran status and prevent the development of unhealthy alcohol use and sedentary behaviors. Meerwijk et al. found that in active-duty members with chronic pain (8% women) who received exercise therapy, those with more than nine visits of exercise therapy prior to separation were less likely to develop alcohol and drug use disorder following the transition, compared with veterans with chronic pain who received no exercise therapy.^49^

Data from a survey study of 22 veterans with substance use disorders (14% WV) suggest that veterans may prefer exercising in Veterans Affairs or gym/fitness club, with a small group, using a combination of self-paced and prescribed, low to moderate exercise intensity, and in one long bout.^48^ In addition, the survey results suggest that veterans may prefer a combination of supervised and unsupervised exercise protocols as well as a recreational/team oriented physical activity program as an addition to their substance use disorder treatment.^48^ Indeed, military sexual trauma and mental health issues are common in WV,^26^ that contributes to increasing risks of alcohol use (as discussed earlier) as well as decreasing levels of physical activity. Littman et al. reported that WV with depression were less likely to meet physical activity guidelines and decreased their physical activity levels after separating from the military.^6^ For those affected by military sexual trauma or PTSD, the authors recommended that exercise protocols be tailored to groups of women with similar age and experiences. In addition, these programs should be delivered using a trauma-sensitive approach, characterized by using welcoming, nonthreatening language, avoidance of physical contact, and the inclusion of elements of mindfulness and dialectical behavioral therapy techniques to support healing and recovery.^41^ Such trauma-informed protocols can provide WV with a comfortable and supportive environment more likely to result in adherence to training and long-term positive outcomes. In addition, Tarlov et al. reported that a large proportion of the U.S. veteran population (no sex reported) lives in areas with fewer food outlets, parks, and commercial fitness facilities.^50^ At-home and digital health interventions, such as Fitbit trackers^38^ and virtual reality,^30^ may solve these barriers for WV, promote their engagement in physical activity, and facilitate intervention adherence.

## Summary

Research on issues affecting WV has increased with the rising number of women joining the armed forces and the poorer health outcomes in WV compared with civilian counterparts. Following military separation, WV have an increased risk for adopting negative health behaviors, including unhealthy alcohol consumption and decreased physical activity. These behaviors are associated with increased risks of CVD and other diseases in WV. In this general narrative review, we found that the majority of research on alcohol use and physical activity mainly focuses on veteran men with a notable lack of studies specifically targeting WVs’ unique needs. The findings from this review suggest that more future studies are needed to conclude the associations between these two lifestyle behaviors in WV. In addition, a handful of studies support the feasibility and the potential role of integrating physical activity intervention into alcohol use treatment for reducing alcohol use in WV. However, the ideal intervention regimen for this distinct and vulnerable group is yet to be defined, raising concerns regarding their health. Continued efforts and research studies are urgently needed to eliminate WV’s health disparities.

## Notes

### Abbreviations Used

AUDIT: Alcohol Use Disorders Identification Test
CVD: cardiovascular disease
HRBS: Health Related Behaviors Survey
PTSD: posttraumatic stress disorder
WV: women veterans
